# Editorial: Natural products targeting oxidative stress and cell death: Treatment potential in metabolic and cardiovascular diseases

**DOI:** 10.3389/fphar.2023.1141878

**Published:** 2023-02-01

**Authors:** Ramoji Kosuru, Yin Cai, Vinod Tiwari

**Affiliations:** ^1^ Versiti Blood Research Institute, Milwaukee, WI, United States; ^2^ Department of Health Technology and Informatics, The Hong Kong Polytechnic University, Kowloon, Hong Kong SAR, China; ^3^ Department of Pharmaceutical Engineering & Technology, Indian Institute of Technology (Banaras Hindu University), Varanasi, Uttar Pradesh, India

**Keywords:** heart failure, oxidative stress, inflammation, natural products, signalling pathway

This Research Topic focused on illuminating the role of natural products in amelioration of oxidative stress, inflammation, and cell death in metabolic and cardiovascular diseases. Although these processes are essential in maintaining homeostatic cell signalling functions under physiological conditions; however, their exacerbation disturbs tissue homeostasis and promotes cell death. The common link for long-term cardiovascular pathologies is oxidative stress, resulted due to an imbalance between the generation of free radical oxygen species (ROS) and endogenous antioxidant enzyme activity. Oxidative stress, inflammation and cell death are implicated in the etiology or pathology of cardiovascular disorders such as chronic heart failure, angiogenesis, atherosclerosis, myocardial infarction, and pulmonary hypertension, accountable for an increase in stroke incidence amongst young populations (Xu et al., 2019). According to the World Health Organization (WHO), cardiovascular diseases are the leading global health concern that leads to morbidity and mortality globally. Approximately 17.9 million people die each year from cardiovascular diseases, accounting for 32% of all deaths worldwide (WHO, 2021). Therefore, it is imperative to carefully investigate the underlying mechanisms involved in the pathogenesis of metabolic and cardiovascular diseases to develop more effective therapies for the prevention of disease progression.

In recent times, several studies have emphasized the importance of bioactive natural compounds for the prevention and treatment of diabetes and cardiovascular diseases. Medicinal plants or natural products are valuable sources of antioxidants that can help fight against detrimental oxidative stress, inflammation, and cell death, which are exacerbated during cardiovascular disease progression. Natural products therapy for the treatment of metabolic and cardiovascular diseases has shown encouraging results, and declined the mortality rate of these patient populations. Although the progress of natural product research on cardiovascular diseases is satisfactory, a huge number of these natural products remain unexplored, and it warrants a more comprehensive understanding of their beneficial effects. The current Research Topic was conducted to explore recent advances in natural product treatment for cardiovascular diseases, in order to provide valuable clues for promising therapeutic targets and new pathological mechanisms. The Research Topic published seven articles, of which two are review articles and five are original contributions. These articles shed a light on the importance of treatment with natural compounds for the mitigation of cardiovascular disorders. The main goal of this editorial is to provide the roadmap for the quality publications that may update the knowledge about the beneficial effect of natural products for the treatment of cardiovascular diseases.

In this Research Topic, you would find two interesting reviews about the potential applications of berberine and herbal medicine in the treatment of cardiovascular diseases. An et al. emphasized the significance of the antioxidant effects of berberine in preventing cardiovascular disease progression in preclinical and clinical settings. They comprehensively discussed the mechanism of action of berberine in preventing cardiac oxidative stress, providing novel insights into berberine usage in the management of heart failure, arrhythmia, myocardial ischemia/reperfusion, and coronary atherosclerosis. Angiogenesis, the formation of new blood vessels from pre-existing blood vessels, plays a crucial part in the amelioration of cardiovascular pathologies, such as ischemic cardiac diseases and stroke. Li et al. comprehensively reviewed the mechanism of action and potential applications of several pro-angiogenic phytoconstituents including ginsenosides from *Panax notoginseng*, astrgalosides, and calycosin from *Radix Astragali*, paeoniflorin from *Radix Paeoniae*, salvianolic acid B from *Salvia miltiorrhiza*, ferulic acid from *Angelica sinensis*, ilexasponin A1 from *Ilex pubescens*, and puerarin from *Radix puerariae* in ischemic heart diseases. They highlighted that these pro-angiogenic phytochemicals ameliorate myocardial ischemic diseases by reducing oxidative stress, inflammation, and cell death. These outcomes aid in the identification of drug candidates from medicinal plants for the treatment of atherosclerotic and ischemic cardiovascular diseases.

To provide new insights for the treatment of heart failure, the molecular signalling mechanisms of different medicinal plants or natural products, are included in this Research Topic. Heart failure is the most debilitating public health concern with high rate of mortality and morbidity globally. In chronic heart failure, oxidative stress and inflammation occur in the myocardium and promote hypertrophic signalling, cardiac cell death, and cause left ventricular dysfunction and arrhythmia (Milinković et al., 2020). To counteract oxidative stress and inflammation in chronic heart failure, Li et al. investigated the importance of Qishen granule (QSG), a clinically approved traditional Chinese medicine in attenuating cardiac inflammation. The authors employed left anterior descending ligation and splenectomy to explore the QSG effect on inflammation induced by the cardio-splenic axis, which dominates the inflammation injury during heart failure. QSG treatment improved cardiac function, inhibited the release of splenic monocytes, and reduced macrophage recruitment and activation in the acute myocardial infarction-induced heart failure mouse model. Furthermore, QSG downregulated Toll-like receptor 4 (TLR4)/MyD88/nuclear factor (NF)-κB p65 inflammatory pathway to counteract cardio-splenic monocyte activation. Therefore, QSG could be a promising anti-inflammatory therapy for the treatment of heart failure and delay the progression of heart failure.

In another study, Zhang et al. revealed the therapeutic mechanism of the Shexiang Tongxin Dropping Pill (STDP), a traditional Chinese Medicine for chronic heart failure. STDP treatment relieved cardiomyocyte hypertrophy, myocardial fibrosis and improved cardiac function in transverse aortic constricted (TAC) mouse, a chronic heart failure mouse model. Using network pharmacology analysis and whole-transcriptome sequencing, the authors revealed that STDP is a “multi-component, multi-target” drug, and shows great potential for the mitigation of chronic heart failure. Interestingly, STDP treatment normalized the 395 imbalanced genes of chronic heart failure in the TAC sugery mouse model. Of note, STDP targets signalling pathways related to fibrosis, energy metabolism, and inflammation by inhibiting transforming growth factor (TGF-β) and ERK/mitogen activated protein kinase (MAPK) pathways. In a similar manner, using the network pharmacology approach, Guo et al. revealed that the calcium signalling pathway is a molecular target for the Danhong injection (DHI) in the prevention of acute myocardial infarction. DHI regulates the calcium signalling pathway, of importance, by inhibiting phospholamban (PLB), sarcoplasmic reticulum Ca^2+^ ATPase (SERCA), and calcium/calmodulin-dependent protein kinase II gamma (CaMK II) protein expression to improve acute myocardial infarction. Additionally, DHI is effective in reducing the key pathological changes of acute myocardial infarction such as the inflammatory cell intrusion, myocardial cell edema, and degeneration in the mouse models. These studies suggest that STDP and DHI are new treatment options for the prevention and treatment of heart failure, which can interfere with the process of cardiovascular diseases from different aspects.

Pulmonary hypertension is a complex and serious condition characterized by high blood pressure in the lung arteries. In a chronic cardiopulmonary dysfunctional state, progressive remodelling of pulmonary arteries and the building up of elevated lung arterial resistance lead to right ventricular failure and death (Simonneau et al., 2019). There are no effective targeted drugs for the treatment of pulmonary hypertension. Wang et al. demonstrated that Dan-Shen-Yin (DSY) granules are effective in ameliorating hypoxia-induced pulmonary hypertension (HPH). 147 potential targets of DSY on HPH were identified based on network pharmacology, of which, oxidative stress (“HIF-1 signaling pathway,” and “response to hypoxia”), inflammation (“response to lipopolysaccharide,” “inflammatory response”), cell proliferation (“negative regulation of the apoptotic process,” and “positive regulation of cell proliferation”), and PI3K-Akt signalling pathway are highly enriched by DSY. In addition, authors demonstrated that DSY targets AKT serine/threonine kinase 1 (AKT1), signal transducer and activator of transcription 3 (STAT3), and hypoxia-inducible factor-1 (HIF-1) proteins to decrease the detrimental effects of HPH, using a hypoxia-induced mouse model. They observed that DSY therapy downregulated proliferating cell nuclear antigen (PCNA) in lung tissues through suppression of the STAT3/HIF-1α and FAK-AKT signalling pathway. Therefore, we can conclude that DSY can prevent the development of HPH and alleviate pulmonary vascular remodelling through multiple pathways.

Hosseini et al. evaluated the protective effect of Rheum turkestanicum on doxorubicin-induced cardiotoxicity due to its substantial *in vitro* antioxidant activity. The central outcome of this study is that Rheum turkestanicum inhibited doxorubicin-induced cardiotoxicity and decreased pathological abnormalities by controlling oxidative stress and antioxidant defenses in the cardiac tissue. The authors suggested that Rheum turkestanicum might be beneficial as a complementary strategy for reducing digoxin-induced cardiotoxicity.

According to these studies, it is hypothesized that reducing oxidative stress, inflammation, and cell death is beneficial in treating cardiovascular disorders such as heart failure and pulmonary hypertension. The current findings are impressive, but further research is needed before natural products may be used to treat metabolic and cardiovascular problems. First, it is very difficult to determine their clinical usefulness due to a scarcity of clinical validation examining these natural compounds’ long-term impact on cardiovascular outcomes. Second, a contentious issue that undermines the favorable therapeutic effects against cardiovascular illnesses is the limited systemic bioavailability of phytochemicals and nutraceuticals. Is it still unclear whether novel drug delivery techniques will increase the systemic bioavailability of these agents? Furthermore investigating any possible pharmacokinetic or pharmacodynamic interactions between natural products and medications provided for CV patients is important for its integral therapy. Natural products should be taken into account as a crucial component of therapy despite these limitations, especially for patients who fall into the high and very high-risk categories because they may assist to reduce residual risk and achieve treatment objectives. We think that the most recent research on a variety of natural products that are highly relevant to the treatment of cardiovascular disorders was published in this Research Topic.
